# Challenges and Strategies for Improving the Regenerative Effects of Mesenchymal Stromal Cell-Based Therapies

**DOI:** 10.3390/ijms18102087

**Published:** 2017-10-02

**Authors:** Silvia Baldari, Giuliana Di Rocco, Martina Piccoli, Michela Pozzobon, Maurizio Muraca, Gabriele Toietta

**Affiliations:** 1 Department of Research, Advanced Diagnostic, and Technological Innovation, Regina Elena National Cancer Institute, via E. Chianesi 53, Rome 00144, Italy; silvia.baldari@ifo.gov.it (S.B.); giuliana.dirocco@ifo.gov.it (G.D.R.); 2 Stem Cells and Regenerative Medicine Laboratory, Foundation Institute of Pediatric Research “Città della Speranza”, corso Stati Uniti 4, Padova 35127, Italy; m.piccoli@irpcds.org; 3 Department of Women’s and Children’s Health, University of Padova, Via Giustiniani 3, Padova 35128, Italy; m.pozzobon@irpcds.org (M.P.); muraca@unipd.it (M.M.)

**Keywords:** anoikis, cell survival, cell therapy, cell transplantation, extracellular vesicles, hypoxia, mesenchymal stromal cells, regenerative medicine

## Abstract

Cell-based therapies have the potential to revolutionize current treatments for diseases with high prevalence and related economic and social burden. Unfortunately, clinical trials have made only modest improvements in restoring normal function to degenerating tissues. This limitation is due, at least in part, to the death of transplanted cells within a few hours after transplant due to a combination of mechanical, cellular, and host factors. In particular, mechanical stress during implantation, extracellular matrix loss upon delivery, nutrient and oxygen deprivation at the recipient site, and host inflammatory response are detrimental factors limiting long-term transplanted cell survival. The beneficial effect of cell therapy for regenerative medicine ultimately depends on the number of administered cells reaching the target tissue, their viability, and their promotion of tissue regeneration. Therefore, strategies aiming at improving viable cell engraftment are crucial for regenerative medicine. Here we review the major factors that hamper successful cell engraftment and the strategies that have been studied to enhance the beneficial effects of cell therapy. Moreover, we provide a perspective on whether mesenchymal stromal cell-derived extracellular vesicle delivery, as a cell-free regenerative approach, may circumvent current cell therapy limitations.

## 1. Introduction

Preclinical investigations have encouraged the development of novel cell therapy approaches to promote tissue regeneration [1]. However, translational studies have demonstrated mixed results [2]. The moderate benefit seen in clinical trials is, at least in part, due to the limited viability of the transplanted cells, regardless of the origin of the donor cells and the degenerative disease under investigation. In fact, up to 99% of grafted cells may die within the first few hours after transplantation, due to the rigors of the microenvironment they encounter upon transplant [3,4]. The cause of rapid death of the transplanted cells is likely to be a combination of different environmental stresses cells face both before and after transplantation and implantation.

Here we review the major obstacles to long-term cell survival at the implantation site that are slowing progress and translational clinical research in the cell therapy field. Moreover, we discuss the multiple strategies that have been used to attempt to enhance cell therapy’s beneficial effects in regenerative medicine, with particular emphasis on mesenchymal stromal cell therapy.

## 2. Challenges to Successful Mesenchymal Stromal Cell Transplantation

Nearly 600 cell therapy clinical studies involving mesenchymal stromal cells (MSCs) are recorded in the National Institutes of Health (NIH) clinical trial registry (Available online: www.clinicaltrials.gov). MSCs have been used for their ability to promote tissue repair and wound healing [5], for immunomodulation [6], and as a vehicle for targeted cancer therapies for their tumor homing properties [7,8,9].

Age and pathological conditions are among the factors affecting the therapeutic potential of cell therapy [10]. In fact, aging and disease are linked to perturbations at the genomic, epigenomic, and proteomic levels [11], which negatively influence MSCs’ functional activities [12]. Cell proliferation and differentiation, paracrine signaling, and the ability to promote injury repair can be deteriorated in MSCs isolated from older subjects, in patients affected by diabetes, obesity, and cardiovascular disorders [10,13,14,15]. Equally, age and disease cause changes in the recipient site in which the cells are administered, possibly attenuating the efficacy of both autologous and allogeneic cell based therapies [16].

The limited success of the majority of the completed protocols underscores the need to minimize massive MSC death after transplant for improving the efficacy of cell transplantation procedures. During the transplantation procedure, MSCs undergo different processes that can potentially affect their performance and be responsible for the high attrition of donor cells upon transplant. In particular, transplanted cell survival may be affected by: (1) anoikis, due to the need to detach anchorage-dependent cells from their substrate for injection and to cellular tensegrity loss after implantation; (2) mechanical stress during the implantation procedure; (3) oxygen and nutrient deprivation, due to low diffusion into poorly vascularized environments; and (4) inflammation-related factors, linked to the possible activation of the host immune response.

### 2.1. Cell–Extracellular Matrix Interactions

Clinical applications of MSCs are based on single cell suspension, in which interactions between cells and the extracellular matrix (ECM) are lost and adhesion signals are downregulated with consequent apoptosis, better defined as anoikis. Such cell death could be limited by preserving cell–cell–ECM contact, as demonstrated by He and colleagues [17]. In this work, embryonic stem cells cultured in Matrigel regained the adhesion molecules, illustrating a long-term engraftment in a murine myocardial ischemia model. These results suggest that ECM not only acts as a spatial and mechanical scaffold but also supports cell adhesion and engraftment. Moreover, there is evidence that cell behavior is the result of a network of extracellular signals, where ECM-released soluble factors can play a pivotal role in either self-renewal or lineage commitment [18,19,20].

Cross-talk among cells, growth factors, and ECM is required for successful tissue regeneration. Manipulating the biological signals produced by ECM mimicking the natural regenerative process could improve the outcome of stem-cell-based therapy, as demonstrated by using hydrogel ECM [21] or adding growth factors with high affinity for ECM [22,23]. In these studies, wound healing was enhanced (see also Section 3.1).

### 2.2. Mechanical Stress

In most cell therapy procedures, cells are re-suspended into a low-viscosity solution, such as saline solution, and then administered topically or systemically using a syringe needle or a catheter [24]. During injection cells are exposed to mechanical stresses, in particular to stretching and shearing forces generated by the extensional and linear flow into the syringe needle or catheter, causing cellular membrane disruption. Some studies estimated that up to 40% of cells are damaged during the injection procedure [25], while others reported a negligible effect [26]. This discrepancy may depend on the cell type and on the method of analysis [27]. Nonetheless, it is conceivable that injected cell viability and function could be significantly improved by optimization of the delivery protocols [28].

### 2.3. Hypoxia and Nutritional Stress

Administration site is of critical importance for the subsequent survival of transplanted cells. Cell based therapies are under investigation for the treatment of pathological conditions such as cardiovascular diseases and wound healing. Consequently, transplantation sites are often characterized by a pro-inflammatory status, reduced pH, and oxidative stress, caused both by inflammation and by the reduction of the arterial blood supply due to damage, constriction, or blocking of blood vessels.

One of the major reasons for early implanted cell death is the lack of oxygenation resulting from delayed revascularization at the site of implantation. Oxygen passive diffusion supports cells at a distance of up to 200 microns from the oxygen source [29]. The usual technique of implantation, consisting of direct injection into a damaged tissue of the maximal number of cells delivered in the minimal number of sites to reduce trauma, results in prolonged exposure of the transplanted cells to hypoxia, further exacerbated by poor oxygen diffusion within the injected cell clump. Transplanted cells are hence subjected to a dramatic transition from the in vitro culture condition, generally characterized by ~20% O_2_, to the anoxic state they face upon transplant [30]. Analogously, after implantation and before vascularization of the transplant may occur, implanted cells rely only on diffusion for nutrient support. Therefore, implanted cells face severe oxidative, acidic, and nutritional stresses upon transplant [31].

The benefits of cell transplantation could be improved by modifying donor cells before transplant to enhance their resistance to hypoxic stress (donor cell preconditioning, refer to Section 3.3) [32,33]. Alternatively, the harsh condition at the site of injection can be adapted in order to support transplanted cell survival (host tissue preconditioning, see Section 3.4) [34].

### 2.4. Immune Response

The vast majority of cell-based therapies apply MSCs, mainly derived from either bone marrow or adipose tissue. The intrinsic low immunogenicity of MSCs, in addition to their immunosuppressive properties, results in reduced immune response after implantation of both autologous and allogeneic MSCs. Nonetheless, to achieve clinically relevant numbers of cells suitable for cell therapy procedures in humans, MSCs need to be expanded in vitro before transplantation. The use of xenobiotic components in tissue culture medium augments the risk of antigen contaminations in cell preparations, with consequent potential activation of the innate immune response leading to acute rejection of transplanted cells [35]. Moreover, MSC systemic administration may trigger an instant blood-mediated inflammatory reaction [36] and complement activation [37] that compromise donor cell survival and function after infusion. Allogeneic MSCs promote a specific cytotoxic T cell response in vitro and the production of antibodies triggering complement-mediated lysis in vivo [38]. In addition, both allogeneic and autologous MSCs can be lysed by activated NK cells [39].

## 3. Strategies for Successful Stem Cell Transplantation

**Figure 1:**
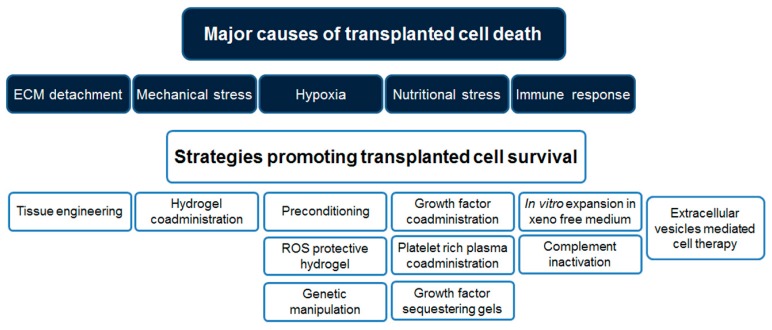
Schematic representation of the major factors limiting cell survival during the transplantation procedure and possible strategies to improve cell-based therapies. Abbreviations: ECM: extracellular matrix; ROS: reactive oxygen species.

How cell therapy exerts its beneficial effects in regenerative medicine procedures has not yet been precisely elucidated. It is likely that transplanted cells promote tissue regeneration through a combination of tissue repopulation and paracrine actions. In any case, the therapeutic effect of cell therapy can be improved by increasing the number of transplanted cells remaining viable and consequently able to promote tissue regeneration at the site of implantation. Therefore, several strategies aiming at counteracting the stress suffered by the cells during the transplantation procedures have been developed [40], as schematically represented in Figure 1 and reviewed in the following sections.

### 3.1. Tissue Engineering: Co-Delivery of Extracellular Matrix Molecules

Interventions and preconditioning enhancing transplanted cell survival and improving cell retention, include co-delivery of extracellular matrix molecules [41]. The tissue engineering approach aims to enhance the survival of exogenous cells, allowing their homing and adaptation before starting their regenerative activity in the transplanted organ. Biomaterial approaches use suitable carriers, serving as synthetic analogs or biologic-derived ECM, to provide a substrate for MSC adhesion, to control cell localization in vivo, and to serve as a scaffold for tissue repair exerted both by exogenous and resident cells [42,43]. Using material-based approaches, it is possible to protect cells from death due to anoikis or inflammatory cell attack, and modulate MSCs’ regeneration abilities. The choice of specific carriers can also strongly affect the performance of MSCs in some applications, because they can help direct the multipotent stromal cell fate towards the desired phenotype [44]. Recently, Ansari and colleagues demonstrated that both the porosity and the elasticity of the hydrogel biomaterial play an important role in dental-derived MSC–immune cell interplay and, therefore, in MSC viability and differentiation. In their work, hydrogel physical properties and microarchitecture regulated the in vivo permeation of pro-inflammatory cytokines and T-lymphocytes, as well as the osteogenic differentiation of MSCs [45]. Similarly, it has been demonstrated that substrate stiffness affects the differentiation process of resident liver stem cells [46].

Biologic scaffolds, such as decellularized tissues, can also enhance MSC engraftment and transplant efficiency by providing a more physiological environment for the cells. Biomaterials can incorporate or mimic ECM function and enhance the survival and differentiation of transplanted cells. In bone regeneration, developing a smooth and open pore scaffold is necessary to enhance MSC adhesion, survival, and function [47,48]. Moreover, biomaterials, in synergy with implanted MSCs, can also promote angiogenesis and enhance tissue-resident progenitor cells proliferation and regeneration by cytokine expression with subsequent activation of macrophages, fibroblasts, and smooth muscle cells [49].

#### Cell-Assisted Lipotransfer

Cell-assisted lipotransfer, consisting of fat grafts supplementation with adipose tissue-derived MSCs [50] before transplantation, has been developed to promote long-term graft retention in regenerative procedures [51]. As MSCs support adipose tissue graft, mixing cells with lipoaspirate might restore a more supporting niche promoting cell survival [52].

### 3.2. Hydrogel Microcarriers to Reduce Mechanical Stress During Cell Administration

Significant MSC loss has been associated with syringe-based administration, due to mechanical stress leading to cell membrane damage, induction of apoptosis, and retention in the delivery device [27]. Moreover, during the procedure, cells may undergo to phenotypic expression changes. Natural and synthetic hydrogels may help to increase the viscosity of the cell suspension during injection, reducing the mechanical forces applied in the procedure [53,54]. An alternative approach to improve cell viability upon injection is represented by cell encapsidation into hydrogels to shield them and reduce damage [25,55].

### 3.3. Preconditioning to Improve Cell Resistance to Stressful Stimuli

Several strategies for preconditioning have been developed to make transplanted cells more resistant to death stimuli following transplantation [56,57,58]. One strategy consists in promoting a broad pro-survival response, through cell exposure to a physical or environmental shock, such as high temperature, hypoxia, anoxia, acidosis, or nutrient deprivation [59] (Table 1). In fact, exposure to sub-lethal conditions allows cells to gradually adapt to changes in their environment, mounting an anti-stress response that activates pro-survival pathways [60]. A more specific approach uses pharmacological modulators of targeted molecules to confer cytoprotective function [61] (Table 2).

#### 3.3.1. Thermal Preconditioning

Heat shock, consisting of incubating the cells at 42 °C for 1–2 h before transplant, has been proven to promote cell survival in transplantation procedures performed in rodents [62,63,68]. This effect is associated with an induction of the expression of heat shock proteins that directly counteract the increased levels of unfolded or denatured proteins working as molecular chaperones and indirectly promoting cell survival by inhibiting apoptotic pathways. Mild heat exposure confers to the cells the transient ability to not only tolerate otherwise lethal temperature elevations, but also to be more resistant to other stress stimuli such as oxidative stress and nutrient withdrawal.

#### 3.3.2. Hypoxic Preconditioning

During the transplantation procedure, cells are exposed to different O_2_ concentrations. MSCs for clinical procedures are conveniently isolated either from the bone marrow or adipose tissue. Oxygen tension in the bone marrow MSC niche is approximately 1–7%, while in adipose tissue it is in the 10–15% range [30]. The first stress occurs when cells are expanded in vitro, generally cultured in ~21% O_2_ atmosphere. Then, after transplantation, MSCs face a hypoxic/anoxic microenvironment due to poor vascularization at the transplant site. Thus, it has been suggested that mitigation of the oxygen shock may enhance the efficacy of hematopoietic stem cell transplant [69]. Consistently, strategies aimed at promoting defense mechanisms against oxidative stress have been developed [70]. For instance, cultivation of MSCs in low oxygen (0.5–3%) may promote increased engraftment via the activation of anti-apoptotic genes including Akt, Bcl-2, and HIF-1α and the upregulation of chemokine receptors such as CXCR4 and CX3CR1 [32,64,71,72,73]. Even anoxic preconditioning has been considered to promote the survival of transplanted MSCs [65]. In addition, preconditioning of MSCs with a low concentration of H_2_O_2_ for a short period has been shown to have a protective effect against more severe and prolonged oxidative stress [74].

#### 3.3.3. Acidic Preconditioning

Ex vivo preconditioning of bone marrow-derived ckit^+^ cells in an acidic (pH 7.0) medium for 24 h prior to transplant was associated with induction of stromal-derived factor-1 (SDF-1) expression, leading to enhancement of therapeutic potential in a mouse model of hindlimb ischemia [66].

#### 3.3.4. Nutrient Deprivation Preconditioning

Cell therapy procedures typically require the administration of a large number of cells [3]. Therefore, before transplantation, MSCs need to be expanded in vitro. To promote cell proliferation and metabolism in vitro, the culture media contain amino acids, vitamins, inorganic salts, glucose, and serum. However, the microenvironment cells encounter after implantation is characterized by poor nutrient support. Diminishing the energy supply before transplant may help cells to gradually adapt to the low energy environment they face after transplant. In fact, reducing metabolic demand by cultivating MSCs in a serum-depleted medium before transplant has been proven to be a simple strategy to induce cellular quiescence [67]. Quiescent MSCs are able to withstand prolonged periods of hypoxia and glucose deprivation in vitro and have enhanced the engraftment rate in vivo after subcutaneous implantation in mice [67].

#### 3.3.5. Pharmacologic Cell Preconditioning

Pharmacologic preconditioning of cells before transplantation is a promising strategy to curtail the massive death of cells after transplantation [61]. Several substances targeting different biological pathways have been used to treat cells before transplantation, in order to increment cell survival in vitro and in vivo. Articles that demonstrate an enhanced cell engraftment in vivo in numerous animal models by different pharmacologic preconditioning strategies have been included in Table 2. Some drugs, such as antioxidants and HIF-1α stabilizers, help cells to cope with increased oxidative stress. Mitochondrial electron transport inhibitors, such as antimycin, have been used to block the activation of mitochondrial death pathways [75]. Other pharmacologic compounds, such as anti-ischemic drugs and K^+^ channel activators [76], have been used to mimic ischemic conditioning. The use of anti-apoptotic drugs has also been described to promote cell survival [77].

### 3.4. Tissue Preconditioning to Make Recipient Site More Receptive to Donor Cells

Tissue preconditioning is a method complementary to donor cell preconditioning. In tissue preconditioning the recipient site of the transplant, instead of the donor cells, is treated before cell administration in order to make the environment more favorable for engraftment [34]. Accordingly, pharmacological modulation by vasodilatory drugs in the recipient site of transplant has been used to enhance therapeutic delivery of adipose tissue-derived MSCs in a mouse model of cardiac repair [92]. Environmental tissue preconditioning can also be performed using physical methods such as the application of a high dose of ultrasounds to the recipient site. For instance, in a clinical trial of bone marrow-derived mononuclear cells transplant in patients with heart failure, significant, albeit modest, benefits have been described when pretreating the recipient site with a low-energy shock wave treatment to promote transplanted cell homing [93]. Viral-mediated gene transfer has also been used to reduce the harsh microenvironment at the site of transplantation, promoting angiogenesis before cell transplant [94]. Moreover, reversible ischemia and reperfusion injury in the liver [95] and heart [96] could increase engraftment, enhancing homing of transplanted MSCs into the ischemic zone.

### 3.5. Cell Co-Transplantation with Active Biomaterials for Oxidative Stress Protection

Cell encapsidation into an injectable anti-oxidant hydrogel [97,98] and the use of hydrogels producing oxygen in a controlled way [99] have been recently designed and proven to reduce cell necrosis induced by hypoxia. This type of biomaterial can, therefore, be used to improve the hostile hypoxic conditions for cell therapy procedures.

### 3.6. Genetic Engineering to Improve Cell Survival

MSCs can be efficiently transduced using both viral and non-viral gene transfer methods [100,101]. Ex vivo genetic manipulation represents an option to reinforce donor cells before transplant [102]. For instance, gene transfer of pro-survival or anti-apoptotic genes, such as protein kinase B (Akt/PBK), B-cell lymphoma-2 (Bcl-2), survivin, and hepatocyte growth factor (HGF) enhanced the survival of MSC in vivo (Table 3). Also, concomitant expression of a pro-survival gene (Akt) and a pro-angiogenic gene (Ang-1) has been proven to promote enhanced cell survival in a mouse model of cardiac injury [103]. Moreover, gene therapy vehicles have been used to promote the expression of some proteins involved in cell adaptation to environmental stress such as heat shock protein 27 [104], or superoxide dismutase 2 to improve oxidative stress resistance [105]. Clearly, both the short- and long-term effects of such genetic manipulations should be carefully evaluated in view of a possibly increased risk of tumorigenesis.

In order to counteract anoikis triggered by extracellular matrix detachment, transplanted cells have been engineered to express genes promoting cell adhesion such as transglutaminase [106] and integrin-linked kinase [107,108].

The therapeutic effect of cell-based therapy for regenerative disorders depends on the number of administered cells reaching the target tissue [109]. MSCs express low levels of molecules such as the homing factor SDF-1 and the CXCR4 and CCR1 receptors, which play a pivotal role in homing. Therefore, strategies to promote the expression in donor cells of proteins involved in homing have been used to enhance the therapeutic efficacy of cell-based therapies [110,111,112,113].

Also, miRNA overexpression has been described as promoting MSCs survival [114,115,116]. In fact, a single miRNA is able to regulate several hundred mRNAs, modulating the gene networks involved in most of the cellular process, including cell survival.

Taken together, several studies have demonstrated that a combination of gene and cell therapy may represent a strategic development in regenerative medicine (Table 3), although confirmation in the clinical setting is required [100].

### 3.7. Providing Nutrient Support to Transplanted Cells

After transplantation, MSCs encounter a progressive and extensive depletion of both oxygen and nutrients. MSCs can survive severe and prolonged hypoxia, as long as an adequate glucose supply is provided [31]. Therefore, supplying nutrients to transplanted cells may reduce the serious metabolic deficit that hinders successful engraftment. As multiple processes seem to be responsible for transplanted cell death, simultaneous targeting of key components of cell survival pathways may be needed. For instance, a pro-survival cocktail containing several factors, including Matrigel to prevent anoikis, inhibitors of mitochondrial death pathways, a K^+^ channel activator, IGF-1, and a caspase inhibitor, has been used to limit cardiomyocyte death after transplantation [127]. In a similar manner, a better engraftment of myogenically converted dermal fibroblasts was obtained using a cocktail containing a combination of pro-survival and anti-apoptotic agents [128]. An alternative approach is represented by co-transplantation of MSCs and platelet-rich plasma, already used in clinical therapy for its high content of growth factors and secreted proteins able to induce the recruitment and proliferation of cells involved in wound healing and tissue regeneration [129].

Also, when loaded into scaffolds, transplanted cells exhibit poor vascularization. Consequently, they are exposed to gradiented nutrient concentrations, mostly limited to the ones present within the scaffold during implantation. Therefore, biomaterials able to sequester and release exogenously added and endogenously produced growth factors have been developed to make trophic support available to transplanted cells [130].

### 3.8. Ex Vivo Expansion in Xeno-Free Media to Reduce the Risk of Immunological Reaction

Although MSCs do not seem to be immunogenic per se, cells expanded in culture media containing xenobiotics may produce immune reactions in patients receiving cell transplant, due to the presence of immunogenic contaminations [131]. Therefore, culture conditions need to be optimized, developing alternative culture protocols for in vitro MSCs expansion in xeno-free media before transplant [35].

### 3.9. Counteracting Complementary Activation

Systemic administration of MSCs has been associated with innate immune response mediated by complement activation that may lead to serious donor cell damage and rejection [36,37]. Therefore, preventing complement induction could improve transplanted cell survival. Accordingly, treatments with complement inhibitors, such as factor H or heparin, have been proposed to locally reduce complement activation on MSCs surface [132,133]. As an alternative approach, transfer into MSCs of the gene encoding for the human cytomegalovirus US2 protein, which is involved in the evasion of cellular antiviral immunity, resulted in protection against lysis induced by complement [134].

### 3.10. The Alternative Approach of Cell-Free Therapy

MSCs exert many of their effects via paracrine signaling [135,136,137,138,139]. Therefore, several investigators are exploring the possibility of replacing the cells with their secretome for therapeutic applications [140]. Bioactive signals produced by MSCs can be secreted as soluble molecules or packed into more complex structures named extracellular vesicles (EVs). EVs are a heterogeneous family of nanoparticles composed of a lipid bilayer and enclosing cytoplasmic components [141,142,143]. EVs are able to transmit signals to target cells by interacting at the cell surface, by internalization, or by fusion with the cell membrane. The EV cargo, including proteins, nucleic acids and lipids, is influenced by cell culture conditions and can be engineered to enhance the expression of desired activities or introduce specific effector molecules [144,145,146,147].

MSC-derived EVs can reproduce some immunomodulatory functions exhibited by their cells of origin, and more recent data suggest that these particles may even exert some distinct and possibly more reproducible immune regulatory effects. In summary, MSC-EVs inhibit both the proliferation and the differentiation of activated B cells, suppressing antibody production [148], and induce the apoptosis of activated T cells while increasing the proliferation of regulatory T cells (Treg) [149]. MSC-EVs also suppress innate immunity, including the activation of NK cells [150] and monocytes [151]. Several preclinical studies in animal models showed that MSC-EVs convey many of the pro-regenerative effects exhibited by their cells of origin. MSC-EVs were able to accelerate skin wound healing [151], improve kidney histology and function following a variety of injuries [152,153], reduce infarct area in ischemic cardiac injury [154,155], and improve brain function following hypoxic-ischemic injury [156].

Since EVs are efficient conveyors of molecular signals that can target specific cell populations, they are being explored as drug carriers for the treatment of cancer and several other diseases [157,158,159]. In particular, the EVs ability to transfer functional mRNA and microRNA allows us to manipulate both the phenotype and the metabolic activity of recipient cells [160,161]. Such novel biological tools could thus be exploited to improve the viability and direct the function of MSCs by conditioning the cells before transplantation.

Some recent reviews discuss in more detail the biology and possible clinical applications of MSC-EVs [162,163,164,165,166]. However, several hurdles need to be addressed in the clinical translation of this potentially new therapeutic tool. First, the classification of the cell secretome as a whole should be defined at the regulatory level. Second, the method of administration is critical, since the systemic distribution of a (somewhat undefined) mixture of bioactive molecules can generate multiple adverse events. Indeed, to our knowledge clinical translation so far has been limited to local applications, such as intradermal injections for the treatment of hair loss [167]. In this respect, EVs could be a more promising therapeutic tool, since they represent a physically distinct fraction of the secretome and seem to convey a definite set of signals with more limited and predictable effects. The good manufacturing practices (GMP) production and release of EVs is less complex compared to living cells, resulting in reduced costs, thus circumventing a major barrier in the diffusion of these innovative treatments [168]. However, EVs are complex biological machines whose function is still largely unknown. The isolation procedures of these nanoparticles are still unsatisfactory, both because current preparations include a significant proportion of contaminants from culture media and because the different classes of EVs (likely conveying different biological activities) are difficult to separate from each other [169,170]. As a result, despite increasing evidence of efficacy in several animal models of disease, clinical applications of MSC-EVs have been limited to a single patient with GVHD [171] and a preliminary trial in patients with kidney failure [172]. Of note, in both cases the EV preparation was not GMP-compliant. Therefore, a series of gaps still needs to be filled to bring these potential therapeutic tools from bench to bedside.

## 4. Conclusions

Cell therapy may represent an attractive strategy for regenerative medicine. However, poor cell survival after transplantation, which is due to a combination of mechanical, cellular, and host factors, limits cell therapy efficacy. We reviewed several strategies that have been developed in preclinical studies to promote the survival of MSCs after transplant. However, before successful translation to the clinic, additional studies should be conducted to more precisely understand whether and how such strategies may affect the in vivo biological activity of transplanted cells. The delivery of MSCs secretome, in particular of extracellular vesicles produced by MSCs, seems to recapitulate the therapeutic benefits observed in MSC-based transplantation. In this context, EVs administration may represent an alternative and innovative strategy for cell-free cell therapy that may circumvent the current limitations associated with poor cell survival upon transplantation.

## Figures and Tables

**Table 1:** Methods of physical and environmental cell preconditioning.

Conditioning Method	Reference
Thermal	[62,63]
Hypoxic	[32,64]
Anoxic	[65]
Acidic	[66]
Nutrient deprivation	[67]

**Table 2:** Pharmacologic mesenchymal cell preconditioning.

Drug Name	Drug Function	Reference
Trimetazidine	Cytoprotective, anti-ischemic	[78,79]
Isoflurane	Cytoprotective	[80]
Erythropoietin	Anti-apoptotic	[77]
Deferoxamine:	HIF-1α stabilizer	[81,82]
Dimethyloxalylglycine	HIF-1α stabilizer	[83]
Antimycin	Mitochondrial inhibitor	[75]
Oxytocin	Anti-oxidant	[84]
Celastrol	Anti-oxidant	[85]
Melatonin	Anti-oxidant	[86,87]
Nicorandil	K^+^ channel activator	[88]
Diazoxide	K^+^ channel activator	[76]
Lipopolysaccharide	TRL4 agonist	[89]
Pioglitazone	PPAR-γ agonists	[90]
NaHS	H_2_S donor	[91]

Abbreviations: HIF-1α: hypoxia inducible factor; PPRA-γ: peroxisome proliferator-activated receptor; TRL4: toll-like receptor.

**Table 3:** Genetic engineering approach.

Cell Type	Gene Name	Gene Function	Reference
MSC	Akt	Anti-apoptotic	[117]
	HGF	Anti-apoptotic	[118]
	Akt and Ang-1	Anti-apoptotic/angiogenesis	[103]
	FGF-2	Pro-survival	[119]
	Hsp27	Pro-survival	[104]
	Survivin	Pro-survival	[120]
	HO-1	Anti-oxidant	[121]
	SOD-2	Anti-oxidant	[105]
	tTG	Promote cell adhesion	[106]
	CCR-1	Promote cell homing	[112]
	CXCR4	Promote cell homing	[113]
	SDF-1	Promote cell homing	[110]
	TERT	Telomerase	[122]
	miR-21, -24, -221	Pro-survival	[115]
	miR-133a	Pro-survival	[114]
	miR-210	Pro-survival	[116]
CM; SMCs; HEP	Bcl-2	Anti-apoptotic	[33,123,124]
SMC	IGF-1	Pro-survival	[125]
EPC	ILK-1	Promote cell adhesion	[107]
CPC	Pim-1	Anti-apoptotic	[126]

Abbreviations: Akt: protein kinase B; Ang-1: angiotensin; CM: cardiomyoblasts; CCR-1: C–C chemokine receptor type 1; CPCs: cardiac progenitor cells; CXCR4: CXC chemokine receptor 4; EPCs: endothelial progenitor cells; FGF-2: fibroblast growth factor; HEP: hepatocytes; HGF: hepatocyte growth factor; Hsp: heat-shock protein; IGF-1 insulin growth factor-1; ILK-1: integrin-linked kinase; OH-1: heme oxygenase; SDF-1: stromal cell-derived factor-1; SMCs: smooth muscle cells; SOD-2: superoxide dismutase; TERT: telomerase; tTG: tissue transglutaminase.

## Abbreviations

ECM	Extracellular matrix
MSCs	Mesenchymal stromal cells
ROS	Reactive oxygen species
EVs	Extracellular vesicles
